# Possible Role of Novel Mitochondrial Subsets in Migraine

**DOI:** 10.3390/life15081273

**Published:** 2025-08-11

**Authors:** Ozgur Yildirim Savran, Meltem Tuncer

**Affiliations:** Department of Physiology, Faculty of Medicine, Hacettepe University, Ankara 06100, Turkey; ozgursavran@hacettepe.edu.tr

**Keywords:** migraine, mitochondrial dysfunction, mitochondrial dynamics, mitochondrial subtypes

## Abstract

Migraine is a complex neurological disorder characterized by recurrent headaches and sensory disturbances. Emerging evidence highlights a critical role for mitochondrial dysfunction in migraine pathophysiology, including impairments in oxidative phosphorylation, disruptions in mitochondrial dynamics, and altered biogenesis. Experimental migraine models—ranging from nitroglycerin-induced attacks to inflammatory stimuli—consistently demonstrate mitochondrial swelling, cristae disruption, decreased ATP production, and increased oxidative stress. These findings are accompanied by the altered expression of key mitochondrial regulators such as PGC-1α, Drp1, and Mfn1. Recent studies have further identified distinct metabolic subtypes of mitochondria, including P5CS-containing subsets, which exhibit unique structural and functional profiles, including cristae loss and reduced ATP synthase expression. Notably, the mitochondrial alterations observed in migraine models show remarkable parallels to those described in P5CS-related mitochondrial subsets. These similarities suggest a potential mechanistic link between metabolic reprogramming within mitochondria and migraine pathogenesis. Understanding the contribution of these newly defined mitochondrial populations could offer novel insights into migraine biology and open new avenues for targeted therapeutic strategies.

## 1. Introduction

Migraine is a recurrent, unilateral, and pulsating headache, often accompanied by nausea, vomiting, photophobia and phonophobia, affecting nearly 1 billion people globally [1]. Migraine significantly impacts various aspects of a person’s life, including career, academic performance, childcare, and social relationships. Additionally, it imposes a substantial economic burden on society due to increased healthcare costs and lost workdays. In the 27 EU countries, the annual cost of migraine has been estimated at EUR 111 billion [2].

Although the exact mechanisms underlying migraine remain unknown, several theories have been proposed, including vascular and inflammatory mechanisms. The vasodilation theory suggests that abnormal vasodilation triggers the activation of the trigeminal nerve; however, this alone is insufficient to fully explain migraine attacks. Another prominent theory is neurogenic inflammation, characterized by vasodilation, plasma protein extravasation, and the release of proinflammatory mediators from mast cells [3]. The activation of nociceptors innervating intracranial blood vessels, cerebral arteries, and sinuses leads to migraine-like headaches. These stimuli are transmitted via the trigeminal nerve to the trigeminal ganglion (TG) and then relayed to the spinal trigeminal nucleus. Subsequently, these signals are projected to higher brain centers, including hypothalamic and thalamic nuclei, and ultimately to multiple cortical regions [4].

Triggers such as skipping meals, physical exertion, dehydration, hypoxia, and sleep deprivation suggest a close link between migraine and metabolic regulation. Several studies have demonstrated alterations in the activity of enzymes encoded by mitochondrial DNA in individuals with migraine. Moreover, the therapeutic efficacy of substances used in migraine management—including glucocorticoids and caffeine—as well as prophylactic agents directly associated with energy metabolism and mitochondrial function, such as riboflavin, coenzyme Q10, and magnesium, further support the notion that disturbances in energy metabolism may play a role in migraine pathophysiology [5].

This connection between metabolic triggers and migraine pathophysiology has led to increasing interest in mitochondrial function. Recent evidence also indicates the existence of mitochondrial subpopulations with distinct metabolic properties—such as pyrroline-5-carboxylate synthase (P5CS)-rich mitochondria—which exhibit altered cristae structure, reduced ATP synthesis, and changes in fission–fusion dynamics [6]. These findings raise the possibility that mitochondrial heterogeneity, particularly involving P5CS-related subsets, may actively contribute to the pathogenesis of migraine. This hypothesis is addressed by outlining key experimental migraine models and evaluating mitochondrial dysfunction—including alterations in morphology, biogenesis, and dynamics—in the context of emerging mitochondrial subtypes.

## 2. Overview of Migraine Models

Although modeling a disease with an unknown etiology is challenging, several methods have been developed to mimic migraine. However, there is no standardized or universally accepted migraine model, and existing models vary in their molecular, behavioral, and treatment response characteristics [7,8].

### 2.1. Dural Stimulation

One of the earliest experimental models of migraine involves the electrical or mechanical stimulation of the dura mater. The electrical stimulation of the dural surface has been shown to induce migraine-like symptoms and is particularly valuable in studying migraine with aura [9]. This stimulation activates meningeal nociceptors via trigeminal afferents projecting to the meninges, leading to the release of vasoactive neuropeptides such as substance P, calcitonin gene-related peptide (CGRP), and neurokinin A. These events contribute to vasodilation and neurogenic inflammation—central mechanisms in migraine pathophysiology [10,11].

Potassium chloride (KCl) is one of the chemical agents used to induce migraine. Similarly to the electrical stimulation of the dura mater, chemical stimulation can also elicit migraine-like attacks with aura-like features [12]. A more recent experimental model involves the application of an ‘inflammatory soup’ to the dural surface. This mixture typically consists of histamine, serotonin, bradykinin, and prostaglandin E2 [13].

Various substances can induce headache through dural irritation, including capsaicin, proinflammatory cytokines, synthetic interstitial fluid with different pH values, and CGRP. These models mimic the neuroinflammatory theory of migraine pathophysiology [14,15].

### 2.2. Algogenic Substances

#### 2.2.1. Calcitonin Gene-Related Peptide (CGRP)

CGRP is a pivotal mediator in trigeminal sensitization and the pathophysiology of migraine. The activation of trigeminal afferents innervating the dura mater leads to the release of CGRP, resulting in vasodilation and increased meningeal blood flow. These neurovascular responses highlight the critical role of CGRP in migraine. Clinical studies have demonstrated elevated CGRP levels in the blood of migraine patients and shown that CGRP infusion can trigger migraine attacks. These findings have prompted the adoption of CGRP in animal-based migraine research. In preclinical studies, CGRP administration via various routes—including intravenous, intraperitoneal, subcutaneous, intracerebroventricular, intrathecal, or dural—has been shown to induce migraine-like pain and associated symptoms [16].

#### 2.2.2. Pituitary Adenylate Cyclase-Activating Polypeptide (PACAP)

PACAP infusion induces vasodilation in meningeal vessels and has been shown to trigger delayed migraine attacks in individuals with migraine [17]. In addition to its vasodilatory effects, PACAP contributes to neuroinflammation and is implicated in the sensitization of trigeminal pathways [18].

#### 2.2.3. Cilostazol

Cilostazol, a phosphodiesterase-3 inhibitor, prevents the breakdown of cyclic adenosine monophosphate (cAMP), resulting in its intracellular accumulation. This process induces the dilation of cranial arteries and can trigger migraine headaches in susceptible individuals [19]. However, the reliability of cilostazol as a model for experimental migraine in laboratory animals remains uncertain. Christensen and colleagues demonstrated that cilostazol induces light sensitivity, grooming behavior, and c-Fos expression in the trigeminal nucleus caudalis. Notably, the behavioral responses observed did not respond to sumatriptan treatment, questioning the validity of this model as a mimic of human migraine [20].

#### 2.2.4. Nitroglycerin (NTG)

NTG administration is one of the most widely used methods for investigating migraine pathophysiology. NTG reliably induces spontaneous migraine attacks in humans and serves as a well-established model in animal studies. NTG is metabolized into nitric oxide (NO), a potent vasodilator. This NO-mediated vasodilation of meningeal blood vessels, along with the activation of neurogenic inflammation, is implicated in the initiation of migraine attacks. Furthermore, NTG administration increases levels of CGRP, which contributes to migraine pathogenesis. NTG also plays a role in activating the trigeminal system and promoting central sensitization, which are key mechanisms underlying migraine development [21].

### 2.3. Medication Overuse

#### Triptans and Opioids

The chronic use of analgesic medications, including triptans, opioids, and nonsteroidal anti-inflammatory drugs (NSAIDs), may induce long-lasting alterations in the trigeminal system, leading to a lowered threshold for headache initiation [22,23].

### 2.4. Genetic Models

Several genetic syndromes associated with migraine, including the subtypes of familial hemiplegic migraine (FHM) (FHM1, FHM2, FHM3, and FHM4), as well as migraine with aura (MA) and familial advanced sleep phase (FASP), have been identified. To study these conditions, migraine models have been developed by generating knockout rodents with mutations in the genes responsible for these syndromes [24].

### 2.5. Specific Channel Activation

#### 2.5.1. ATP-Sensitive Potassium (KATP) Channels

Human studies have demonstrated that the opening of KATP channels with agonists such as levcromakalim can trigger migraine attacks in individuals with a history of migraine [25]. Theoretically, the activation of KATP channels would induce hyperpolarization, which could counteract the hyperexcitability typically associated with migraine attacks. Furthermore, some studies have suggested that KATP channel activation exerts antinociceptive effects. However, recent hypotheses propose that the opening of KATP channels in vascular smooth muscle cells may lead to the depolarization of nearby nerve endings, thereby eliciting headache-like responses [26]. Animal studies have shown that levcromakalim administration induces migraine-like headaches and associated behavioral changes [26,27]. Notably, although levcromakalim rapidly elicits migraine-like responses, it does not lead to an increase in c-Fos expression, a key marker of cortical spreading depression (CSD) [27].

#### 2.5.2. Transient Receptor Potential Ankyrin 1 (TRPA1)

The blockade of the TRPA1 channel has been shown to inhibit the induction of CGRP, whereas the activation of the TRPA1 channel increases CGRP release in the trigeminal ganglion. The administration of the TRPA1 agonist umbellulone has been observed to induce headache and migraine-like pain responses. Furthermore, TRPA1 activation is thought to contribute to the development of CSD [28,29].

### 2.6. Hormone Manipulation

It is well established that females are more susceptible to migraine, suggesting a role for sex hormones—particularly estrogen—in migraine pathophysiology. Experimental studies have shown that ovariectomized rats treated with 17-beta-estradiol exhibit increased susceptibility to CSD and display migraine-like pain behaviors, supporting the involvement of estrogen in migraine mechanisms [30].

### 2.7. Optogenic Model

In transgenic mice expressing channelrhodopsin-2, focal illumination through the intact skull induces an increase in local extracellular potassium levels, thereby triggering CSD. This optogenetic approach offers a non-invasive and highly controllable method for modeling migraine in experimental settings [31].

## 3. Mitochondrial Dysfunction in Migraine

Mitochondria are essential organelles responsible for cellular energy production via oxidative phosphorylation (OXPHOS). This process involves four protein complexes embedded in the inner mitochondrial membrane, along with two mobile electron carriers, which together constitute the electron transport chain (ETC). Electrons are transferred along the ETC, generating a proton motive force across the inner mitochondrial membrane as protons are pumped into the intermembrane space. This electrochemical gradient drives ATP synthesis by complex V (ATP synthase) [32].

Mitochondrial homeostasis is a dynamic and tightly regulated process involving fission, fusion, biogenesis and mitophagy. Damaged mitochondria can be restored through fusion with neighboring healthy mitochondria. However, persistent oxidative stress or mitochondrial DNA (mtDNA) damage may impair fusion, leading to mitochondrial fragmentation and the selective removal of dysfunctional mitochondria via mitophagy. Mitochondrial fusion enables the merging of mitochondrial membranes and is mediated by outer mitochondrial membrane (OMM) proteins mitofusin 1 and 2 (Mfn1, Mfn2), as well as the inner mitochondrial membrane (IMM) protein optic atrophy protein 1 (OPA1). The loss of Mfn or OPA1 has been associated with decreased respiratory function, and defective OPA1 processing in dysfunctional mitochondria may promote their segregation from the mitochondrial network, targeting them for degradation before they can release pro-apoptotic molecules. In contrast, mitochondrial fission is primarily regulated by the cytosolic GTPase dynamin-related protein 1 (Drp1), which translocates to the mitochondrial surface to constrict and divide the membrane. Fission serves to isolate irreversibly damaged or depolarized mitochondria that have lost their fusion capacity, thereby facilitating their elimination through mitophagy and maintaining overall network quality [33].

The biogenesis of new mitochondria is primarily controlled by the transcriptional coactivator peroxisome proliferator-activated receptor gamma coactivator-1 alpha (PGC-1α). PGC-1α activates several nuclear transcriptional programs via nuclear respiratory factors (NRF1 and NRF2), estrogen-related receptors (ERRs), and peroxisome proliferator-activated receptors (PPARs), contributing to the replication of mtDNA, the expression of respiratory chain components, and mitochondrial number and function. Although PGC-1α knockout mice exhibit mitochondria with relatively preserved morphology under baseline conditions, mitochondrial dysfunction becomes apparent under physiological stress, along with a disrupted expression of nuclear-encoded respiratory proteins. Deficiencies in NRF1 and NRF2 have been shown to result in reduced mtDNA content, the loss of mitochondrial membrane potential, respiratory chain defects, and impaired cell cycle progression due to insufficient mitochondrial capacity [34].

Both clinical and preclinical studies have increasingly implicated mitochondrial dysfunction in migraine. Approximately 50% of patients with mitochondrial disease report experiencing migraine [35]. Furthermore, impaired mitochondrial function has been observed even in migraine patients without a diagnosed mitochondrial disorder. These impairments include reduced energy production, increased oxidative stress, and disrupted mitochondrial biogenesis [36,37]. While these findings point to global mitochondrial impairment, recent advances suggest that specific mitochondrial subtypes—rather than a uniform mitochondrial population—may be particularly vulnerable or pathologically active in migraine. This emerging perspective necessitates a closer examination of experimental models to evaluate mitochondrial dynamics in detail.

## 4. Animal Studies Investigating the Role of Mitochondria in Migraine

A literature search was conducted on 21 March 2025, using the PubMed database with the keywords “migraine” and “mitochondria.” Filters were applied to exclude review articles and to restrict results to studies involving non-human animals. This search yielded 21 articles published between 1996 and the date of the search. Following title, abstract, and full-text screening, seven studies were excluded for the following reasons: four were unrelated to migraine (e.g., studies on chronic myeloid leukemia, Wolfram syndrome, myoblasts, or unrelated cellular models), one did not assess mitochondrial function despite initial relevance, one was conducted by the same research group using a similar methodology and reporting overlapping findings with a previously included study—therefore excluded to avoid redundancy in interpretation—and one was excluded due to a lack of full-text access. A summary of mitochondrial alterations observed in various experimental migraine models is presented in Table 1.

### 4.1. Mitochondrial Function in Nitroglycerin (NTG)-Induced Migraine

Several studies have employed the NTG-induced migraine model to investigate the role of mitochondrial dysfunction in migraine pathophysiology.

Wang et al. analyzed NTG-induced migraine in Sprague Dawley rats using Western blotting, electron microscopy, and mitochondrial membrane potential (MMP) assays in the trigeminal nucleus caudalis (TNC), as well as immunohistochemistry, and reactive oxygen species (ROS) and ATP assays in the medulla oblongata. The study reported a reduction in MMP, decreased ATP production, elevated ROS and serum nitric oxide (NO) levels, and the upregulation of neuronal nitric oxide synthase (nNOS), TRPA1, interleukin-1β (IL-1β), and nuclear factor kappa B (NF-κB). Additionally, mitochondrial swelling and cristae disruption were observed [38].

Similarly, Vafei et al. assessed oxidative stress markers in the frontal cortex and found increased oxidative stress along with a significant reduction in ATP levels in Wistar rats, indicating mitochondrial dysfunction [39].

Xie at al. employed a multi-modal approach, analyzing brain tissues including the thalamus, hypothalamus, periaqueductal gray (PAG), trigeminal ganglion (TG), and trigeminocervical complex (TCC). Proteomic analysis, ATP quantification from isolated brain mitochondria, an RT-PCR of the thalamus, and a transmission electron microscopy (TEM) of the ventral posteromedial thalamic nucleus were conducted in C57BL/6J mice. The results demonstrated impaired mitochondrial Complex I activity, reduced NADH and ATP levels, elevated malondialdehyde (MDA) concentrations, and increased mitochondrial fragmentation, suggesting enhanced fission activity [40].

Barbosa et al. conducted cortical metabolic assessments and reported increased glucose uptake in the hypothalamus and inferior colliculus of NTG-treated Wistar rats. However, only minor changes in PGC-1α levels were observed [41].

Li et al. demonstrated mitochondrial biogenesis impairment in Sprague Dawley rats by showing a decrease in mitochondrial DNA (mtDNA) copy number, reduced expression of PGC-1α, mitochondrial transcription factor A (TFAM), and peroxisome proliferator-activated receptor gamma (PPARγ). Furthermore, ATP production and MMP were significantly reduced in the spinal trigeminal nucleus. The pro-apoptotic marker Bax was upregulated, while the anti-apoptotic marker Bcl-2 was downregulated, suggesting activation of mitochondrial-dependent apoptotic pathways [42].

These findings—particularly the cristae disruption, mitochondrial swelling, reduced ATP synthesis, and the altered expression of fission and apoptotic markers—are consistent with the emerging concept of metabolically distinct mitochondrial subtypes characterized by impaired structure and energy production. Notably, several of these features—including cristae loss and ATP depletion—mirror the structural and functional abnormalities reported in P5CS-containing mitochondria (see Section 5).

### 4.2. Mitochondrial Function in Dural Stimulation-Induced Migraine

#### 4.2.1. KCl-Induced Cortical Spreading Depression (CSD)

CSD, a key feature of migraine with aura, has been used to investigate mitochondrial alterations associated with migraine.

Sword et al. employed in vivo two-photon imaging followed by quantitative serial section electron microscopy (ssEM) to examine mitochondrial morphology in the brains of C57BL/6J mice. The analysis revealed significant mitochondrial fragmentation, characterized by an increased presence of both tubular and globular mitochondria. Tubular mitochondria were notably shortened, and mitochondrial swelling was evident, as reflected by an increased mitochondrial diameter [43].

Li et al. assessed mitochondrial respiratory function in Sprague Dawley rats using mitochondria isolated from the cerebral cortex following KCl-induced CSD. The study demonstrated a reduction in state 3 respiration (reflecting maximal ATP-generating capacity) and an increase in state 4 respiration (indicating proton leak across the inner mitochondrial membrane). These alterations resulted in mitochondrial uncoupling, as evidenced by a reduced respiratory control ratio (RCR; state 3/state 4), indicating impaired mitochondrial efficiency in the migraine group [44].

The observed mitochondrial inefficiency and structural alterations—including fragmentation and swelling—closely resemble features associated with stress-induced mitochondrial subtypes, which exhibit compromised bioenergetic and morphological integrity (see Section 5).

#### 4.2.2. Inflammatory Soup-Induced Migraine

The inflammatory soup model, which involves the dural application of a proinflammatory mixture, has been widely used to investigate migraine pathophysiology, including mitochondrial dysfunction.

Shan et al. evaluated mitochondrial function in the trigeminal nucleus caudalis (TNC) of C57BL/6 mice subjected to inflammatory soup administration. The migraine group showed reduced mitochondrial membrane potential and ATP levels, alongside increased ROS and MDA levels. A decline in PGC-1α and TFAM expression was observed, indicating impaired mitochondrial biogenesis. Markers of mitochondrial fission, including Drp1 and Fis1, were upregulated, while Mfn2 remained unchanged. Morphologically, mitochondria were smaller, swollen, and contained fewer cristae. Impaired mitophagy was suggested by increased p62 and decreased Pink1 expression, while Parkin and Beclin1 levels were unchanged. Furthermore, sirtuin 3 (SIRT3) expression was significantly reduced [45].

Dong et al. investigated mitochondrial morphology in the trigeminal ganglia of Sprague Dawley rats. Mitochondria appeared small, fragmented, and asymmetrical, with disrupted ultrastructure, reduced cristae, and vacuolization. Drp1 expression was elevated, while Mfn1 expression was decreased. Additionally, mtDNA and mRNA levels of PGC-1α, NRF1, NRF2, and TFAM were significantly reduced, supporting the presence of impaired mitochondrial biogenesis [37]. The mitochondrial fragmentation, cristae abnormalities, and reduced biogenesis in this model strongly resemble the features attributed to P5CS-enriched mitochondrial populations.

Liang et al. reported that the administration of an inflammatory soup to Sprague Dawley rats led to decreased sirtuin 1 (SIRT1) expression in the TNC, which was associated with the downregulation of TFAM, NRF1, and NRF2. Correspondingly, ATP production and mitochondrial membrane potential were diminished, mtDNA expression was reduced, and mitochondrial swelling with disrupted cristae was observed [46].

The observed impairments in mitochondrial biogenesis, cristae structure, and dynamics align with patterns described in functionally specialized mitochondrial populations that emerge under metabolic stress, as discussed in Section 5.

Fried et al. performed a detailed analysis of mitochondrial respiration parameters—including basal respiration, ATP turnover, proton leak, maximal respiration, and non-mitochondrial respiration—in the brains of Sprague Dawley rats. The study demonstrated a significant reduction in both the spare respiratory capacity and oxygen consumption rate, suggesting functional mitochondrial impairment in this migraine model [47].

### 4.3. Mitochondrial Function in Pituitary Adenylate Cyclase-Activating Polypeptide (PACAP)-Induced Migraine

Takacs-Lovasz et al. explored the effects of PACAP stimulation on cultured rat TG neurons. PACAP activation was associated with increased protein kinase A activity and transient receptor potential vanilloid 1 (TRPV1) channel activity, both contributing to enhanced nociceptive signaling. Evidence of mitochondrial dysfunction was observed at the molecular level, including the downregulation of mitochondrial proteins such as Complex I B6 subunit, Fbl, Fhl2, Slc25a5, and Tomm6. Conversely, several genes were upregulated, including Cenpb, Gnal, Hsp90aa1, Hmga1, Tomm70, Gnai1, and Tomm34, suggesting compensatory or stress-related mitochondrial responses [48].

### 4.4. Mitochondrial Function in Genetic Models of Migraine

Bawa and Abbott assessed mitochondrial function in a genetic mouse model of migraine carrying a mutation in the Cav2.1 voltage-gated calcium channel, which is implicated in Familial Hemiplegic Migraine type 1 (FHM1). In cerebellar granule cells from transgenic mice, MMP was significantly reduced, indicating mitochondrial dysfunction. However, no significant changes were observed in cytochrome c expression or cardiolipin content, suggesting selective impairment in mitochondrial energetics rather than global mitochondrial damage [49].

### 4.5. Mitochondrial Function Following Facial Capsaicin Application

Shibata et al. investigated mitochondrial alterations in the TG of C57BL/6 mice following the facial application of capsaicin, a TRPV1 agonist. Mitochondria in TG neurons exhibited swelling, cristae disorganization, and a reduction in mitochondrial number, indicating structural and functional mitochondrial impairment. In parallel, the expression of cytochrome c oxidase subunit IV (COX IV) was elevated, along with increased mRNA levels of Mic60 (also known as mitofilin) and VDAC1, pointing to a potential stress-induced mitochondrial response [50]. Such responses, including the loss of cristae and mitochondrial swelling, are reminiscent of the ultrastructural changes seen in P5CS-containing mitochondria.

## 5. Metabolic Subtypes of Mitochondria

Recent studies have proposed a functional classification of mitochondrial metabolic enzymes into distinct subtypes based on gene expression clustering. According to Ryu et al. (2024) [6], enzymes involved in mitochondrial metabolism are grouped into three major clusters: the first encompasses enzymes of the tricarboxylic acid (TCA) cycle, the second includes proteins involved in amino acid biosynthesis, and the third consists of enzymes associated with one-carbon metabolism. A pivotal enzyme that interlinks all three clusters is pyrroline-5-carboxylate synthase (P5CS), highlighting its central role in coordinating mitochondrial metabolic pathways [6].

Under stress conditions, P5CS aggregates into puncta-like structures within mitochondria. Mitochondria containing P5CS filaments display a significant decrease in ATP5A1 and ATP5B protein levels, leading to diminished ATP synthesis. Despite maintaining MMP and supporting reductive biosynthetic processes through its enzymatic activity, P5CS-containing mitochondria exhibit a complete loss of cristae structures. Notably, critical proteins required for cristae formation—MIC60, ATP5I, and OPA1—are absent in these mitochondria [6].

**Figure 1:**
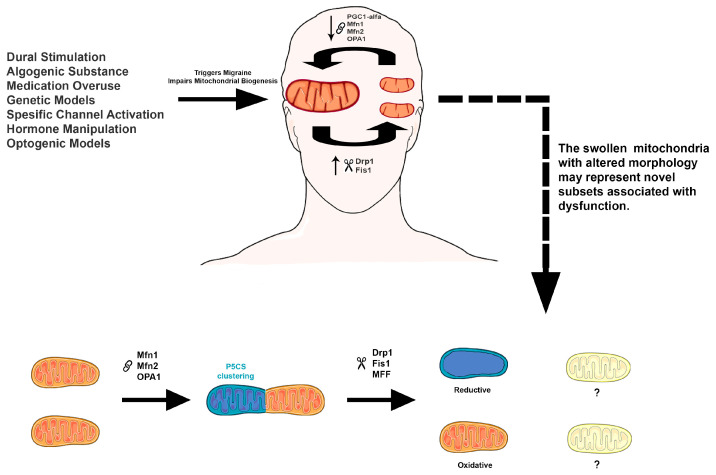
A summary of mitochondrial dysfunction and metabolic subtypes in migraine models. Experimental migraine models—including nitroglycerin, cortical spreading depression, inflammatory stimuli, and PACAP—consistently show disruptions in mitochondrial biogenesis, membrane potential, ATP production, and cristae integrity. These alterations are associated with imbalances in mitochondrial fission (e.g., Drp1, Fis1) and fusion (e.g., Mfn1, Mfn2, OPA1). Notably, such features show striking similarity to those reported in P5CS-containing mitochondrial subsets, which exhibit cristae loss, reduced ATP synthase levels, and altered dynamics. Question marks (?) in the schematic indicate hypothesized or currently unconfirmed mechanistic links between migraine-induced mitochondrial alterations and P5CS-related metabolic subtypes. Upward arrows (↑) indicate increased expression or activity, while downward arrows (↓) indicate decreased expression or activity. The scissors icon represents mitochondrial fission-inducing processes, while the chain icon indicates pathways promoting mitochondrial fusion. This visual summary highlights potential mechanistic overlap and areas for future investigation. Image adapted from Servier Medical Art “https://smart.servier.com/ (accessed on 7 June 2025)”, licensed under CC BY 4.0 (https://creativecommons.org/licenses/by/4.0/).

Mitochondrial fusion dynamics are also impacted by the presence of P5CS. A reduction in mitofusin proteins (Mfn1 and Mfn2) impairs the segregation of P5CS-rich mitochondria from those that harbor functional ATP synthase complexes, thereby limiting the enhancement of oxidative phosphorylation (OXPHOS) capacity. Additionally, decreased levels of Drp1 lead to downstream reductions in mitochondrial fission factor (MFF), Fis1, mitochondrial elongation factor 1 (Mief1), and mitochondrial elongation factor 1 (Mief2)—proteins essential for mitochondrial fission. These disruptions result in aberrant mitochondrial morphology characterized by elongated, swollen, and bulbous structures. Interestingly, Drp1-deficient cells do not exhibit significant impairments in global OXPHOS function; however, they show reduced proline levels, implicating altered amino acid metabolism as a consequence of disrupted mitochondrial segregation and morphology [6]. Figure 1 summarizes the major findings from experimental migraine models in relation to P5CS-containing mitochondrial subsets.

Mitochondrial subtypes enriched in P5CS exhibit key features such as cristae disruption, reduced ATP production, altered fission–fusion dynamics, and mitochondrial swelling—findings that closely resemble those observed in multiple experimental migraine models (see Section 4). These similarities suggest that P5CS-containing mitochondria may play an active role in migraine pathophysiology, rather than representing a general response to cellular stress. Understanding how these metabolically distinct mitochondrial populations contribute to migraine could help identify new mechanisms and potential treatment targets. In this context, mitochondrial dynamics may further contribute to the observed heterogeneity. Several experimental migraine models have reported an increased expression of Drp1 and morphological evidence of mitochondrial fragmentation, particularly in brain regions involved in migraine processing such as the trigeminal ganglion (TG), trigeminal nucleus caudalis (TNC), and periaqueductal gray (PAG). However, alterations in fusion-related proteins—including Mfn1, Mfn2, and OPA1—appear to be less consistently described and may vary depending on the model and region examined.

These changes in fission–fusion balance may reflect compensatory responses aimed at maintaining mitochondrial quality under stress. Nevertheless, persistent or excessive fragmentation has been linked to reduced ATP production, increased oxidative stress, and enhanced apoptotic signaling, all of which may contribute to neuronal dysfunction in migraine. Further studies are needed to determine whether these alterations are protective or deleterious and how they relate to metabolically specialized mitochondrial subsets such as those enriched in P5CS.

Although emerging evidence suggests intriguing parallels between P5CS-containing mitochondria and the structural and functional changes observed in migraine models, several key questions remain regarding the nature, regulation, and disease relevance of these mitochondrial subtypes. The mitochondrial localization of P5CS is determined by its targeting sequence and oligomerization domains, which enable its assembly into filamentous structures. These filaments are not static; rather, they respond dynamically to metabolic cues. Under conditions of increased oxidative phosphorylation—such as culture in galactose medium or stimulation with D-lactate—P5CS forms filaments that segregate into a distinct subpopulation of mitochondria. In contrast, nutrient deprivation (especially amino acid starvation) or oxidative stress modulation (e.g., NAC treatment) disrupts filament formation and leads to a diffuse mitochondrial distribution, without altering P5CS expression levels. This suggests that filament assembly is regulated post-translationally and is sensitive to both energy demand and redox status [51].

The resulting mitochondrial subpopulations enriched either in P5CS or ATP synthase exhibit distinct functional roles. P5CS-containing mitochondria display elevated membrane potential and support reductive biosynthesis, particularly proline production, whereas ATP synthase-rich mitochondria maintain high oxidative phosphorylation capacity. The formation and maintenance of these specialized domains depend on intact mitochondrial fusion and fission dynamics. The loss of MFN1/2 or DRP1 disrupts their segregation and selectively impairs either proline biosynthesis or ATP generation [6]. Furthermore, disease-associated mutations abolish filament formation and mitochondrial compartmentalization despite preserved enzymatic function, reinforcing the structural importance of P5CS oligomerization [51].

Notably, the spatial separation of P5CS and ATP synthase has been demonstrated not only in vitro but also in vivo. In tissue sections from human pancreatic ductal adenocarcinomas, P5CS filaments were observed in metabolically stressed tumor cells but not in adjacent normal tissue. These findings support a model in which P5CS actively contributes to the structural and metabolic diversification of mitochondria under stress. Techniques such as TurboID proximity labeling, high-resolution microscopy, and targeted metabolic perturbation have been instrumental in identifying and characterizing these subtypes [6]. However, further studies are needed to clarify their molecular composition, regulatory mechanisms, and potential involvement in the pathophysiology of migraine and other disorders characterized by mitochondrial dysfunction.

## 6. Conclusions

Compelling evidence from animal studies indicates that mitochondrial morphology is significantly altered in migraine models [38,40,43,45]. Concurrently, impairments in ATP production observed in these models provide evidence of underlying changes in mitochondrial energy metabolism [38,39,40,42].

Similar metabolic disturbances have been reported by Ryu et al. (2024) [6], raising the question of whether the mitochondrial changes observed in migraine may be related to the metabolic subsets they identified. This potential overlap warrants further investigation [6].

When mitochondrial dynamics are examined, migraine models frequently exhibit the upregulation of fission-related genes such as Drp1 and Fis1, along with the downregulation of fusion-related genes like Mfn1 [37,45]. In contrast, Ryu et al. found reduced Drp1 expression and impaired mitochondrial fission in P5CS-containing mitochondria, along with decreased levels of Mfn1 and Mfn2 [6]. These findings suggest a complex interplay between mitochondrial metabolic subtypes and dynamic remodeling processes in migraine.

Human studies have further hinted at metabolic dysfunction in migraine. Reduced serum proline levels in migraine patients [52] may indicate P5CS pathway involvement, while elevated glutamate levels [53] could reflect broader mitochondrial metabolic disturbances, potentially influencing excitatory neurotransmission and migraine pathogenesis.

Recent studies have demonstrated that peripheral blood mononuclear cells (PBMCs) may mimic certain brain conditions, suggesting their potential as a non-invasive source of biomarkers [54]. PBMC analyses in patients with migraine have revealed alterations in inflammatory and metabolic pathways, which are believed to be associated with mitochondrial dysfunction. These findings support the notion that PBMC-based biomarkers could aid in identifying individuals with a predisposition to migraine.

In this context, several studies have investigated migraine-associated microRNA (miRNA) expression profiles. Specific miRNA subsets have been linked to migraine-related mechanisms, including alterations triggered by photophobia, the dysregulation of glutamate metabolism, changes in inflammatory markers, and miRNA-mediated effects on mitochondrial function [55]. These efforts aim to establish a migraine-specific miRNA signature.

Furthermore, energy metabolism-related metabolites such as lactate and succinate—which have been found to be elevated in the plasma of migraine patients—are being studied in conjunction with PBMC transcriptomic data to detect changes during both migraine attacks and interictal periods [56]. Identifying novel mitochondrial subtypes and elucidating their role in migraine pathophysiology may contribute to the development of more specific and non-invasive diagnostic strategies in the future.

Overall, the mitochondrial alterations observed in migraine—including cristae disruption, swelling, diminished ATP production, and dysregulated fission/fusion dynamics—mirror those found in P5CS-linked metabolic subtypes. These parallels suggest that migraine-associated mitochondrial dysfunction may converge on pathways involving P5CS, offering a novel perspective on migraine pathophysiology. Further investigations into the role of P5CS and related metabolic regulators may uncover new therapeutic avenues targeting mitochondrial function in migraine.

Future studies should explore whether P5CS-containing mitochondrial populations define a unique, potentially targetable metabolic vulnerability in migraine. Identifying such subsets could open up novel therapeutic avenues, particularly for treatment-resistant phenotypes.

## Figures and Tables

**Table 1:** Summary of mitochondrial alterations reported across experimental migraine models.

Model	Species	Tissues	Key Findings	Research Team (Year)
Nitroglycerin	Sprague Dawley rats	Medulla oblongata, trigeminal nucleus caudalis (TNC)	Reduced mitochondrial membrane potential (MMP) Decreased ATP production Increased ROS levels Mitochondrial swelling and cristae disruption	Wang et al. (2025) [38]
	Wistar rats	Frontal cortex	Elevated oxidative stress Decreased ATP levels	Vafaei et al. (2024) [39]
	C57BL/6J mice	Thalamus, hypothalamus, periaqueductal gray (PAG), trigeminal ganglion (TG), trigeminocervical complex (TCC)	Altered Complex I activity Decreased ATP production Increased mitochondrial fragmentation (fission)	Xie et al. (2023) [40]
	Wistar rats	Hypothalamus, inferior colliculus	Increased glucose uptake in hypothalamus and inferior colliculus Minor changes in PGC1α levels	Barbosa et al. (2023) [41]
	Sprague Dawley rats	Spinal TN	Decreased mtDNA copy number Reduced PGC1α, TFAM, PPARγ Decreased ATP and MMP Increased Bax, decreased Bcl-2	Li et al. (2016) [42]
KCl-Induced Cortical Spreading Depression	C57BL/6J mice	Brain	Mitochondrial fragmentation Shorter tubular mitochondria Mitochondrial swelling	Sword et al. (2024) [43]
	Sprague Dawley rats	Cerebral cortex	Decreased state 3 respiration Increased state 4 respiration Lower respiratory control ratio (RCR)	Li et al. (2011) [44]
Inflammatory Soup	C57BL/6J mice	TNC	Reduced MMP and ATP Increased ROS and MDA Decreased PGC1α and TFAM Increased Drp1 and Fis1 (enhanced fission) Smaller, swollen mitochondria with fewer cristae Impaired mitophagy (increased p62, decreased Pink1)	Shan et al. (2023) [45]
	Sprague Dawley rats	TG	Small, fragmented mitochondria with altered ultrastructure Increased Drp1, decreased Mfn1 Reduced mtDNA, PGC1α, NRF1, NRF2, TFAM mRNA	Dong et al. (2017) [37]
	Sprague Dawley rats	TNC	Decreased SIRT1, TFAM, NRF1, NRF2 Reduced ATP and MMP Decreased mtDNA Mitochondrial swelling and disrupted cristae	Liang et al. (2021) [46]
	Sprague Dawley rats	TNC	Decreased spare respiratory capacity Reduced oxygen consumption rate	Fried et al. (2014) [47]
PACAP stimulation	Cultured rat TG neurons	TG neurons	Downregulation of Complex I B6 subunit, Fbl, Fhl2, Slc25a5, Tomm6 Upregulation of Cenpb, Gnal, Hsp90aa1, Hmga1, Tomm70, Gnai1, Tomm34	Takacs-Lovasz et al. (2022) [48]
Genetic Model	Cav2.1 transgenic mice	Cerebellar granule cells	Reduced MMP	Bawa and Abbott (2008) [49]
Facial Capsaicin Application	C57BL/6 mice	TG neurons	Mitochondrial swelling, cristae loss, reduced mitochondrial number Increased COX IV, Mic60/Mitofilin mRNA, VDAC1	Shibata et al. (2020) [50]

## Abbreviations

The following abbreviations are used in this manuscript:

ATP	Adenosine Triphosphate
cAMP	Cyclic Adenosine Monophosphate
Cenpb	Centromere Protein B
CGRP	Calcitonin Gene-Related Peptide
COX IV	Cytochrome c Oxidase Subunit IV
CSD	Cortical Spreading Depression
Drp1	Dynamin-Related Protein 1
ERRs	Estrogen-Related Receptors
ETC	Electron Transport Chain
FASP	Familial Advanced Sleep Phase
Fbl	Fibrillarin
Fhl2	Four and a Half LIM Domains Protein 2
FHM	Familial Hemiplegic Migraine
Fis1	Fission 1 Protein
Gnai1	Guanine Nucleotide-Binding Protein G(I) Subunit Alpha 1
Gnal	Guanine Nucleotide-Binding Protein G(olf) Subunit Alpha
Hmga1	High Mobility Group AT-Hook1
Hsp90aa1	Heat Shock Protein 90 Alpha Family Class A Member 1
IL-1β	Interleukin-1 Beta
IMM	Inner Mitochondrial Membrane
KATP	ATP- Sensitive Potassium Channel
KCl	Potassium Chloride
MA	Migraine with Aura
MDA	Malondialdehyde
MFF	Mitochondrial Fission Factor
MFN1	Mitofusin 1
MFN2	Mitofusin 2
Mief1	Mitochondrial Elongation Factor 1
Mief2	Mitochondrial Elongation Factor 2
MMP	Mitochondrial Membrane Potential
mtDNA	Mitochondrial DNA
NADH	Nicotinamide Adenine Dinucleotide (Reduced Form)
NF-κB	Nuclear Factor Kappa-Light-Chain-Enhancer of Activated B Cells
nNOS	Neural Nitric Oxide Synthase
NO	Nitric Oxide
NRF1	Nuclear Respiratory Factor 1
NRF2	Nuclear Respiratory Factor 2
NSAIDs	Non-Steroidal Anti-Inflammatory Drugs
NTG	Nitroglycerin
OMM	Outer Mitochondrial Membrane
OPA1	Optic Atrophy 1 Protein
OXPHOS	Oxidative Phosphorylation
P5CS	Pyrroline-5-Carboxylate Synthase
PACAP	Pituitary Adenylate Cyclase-Activating Polypeptide
PAG	Periaqueductal Graey
PBMC	Peripheral Blood Mononuclear Cells
PGC-1α	Peroxisome Proliferator-Activated Receptor Gamma Coactivator 1-Alpha
PPARs	Peroxisome Proliferator-Activated Receptors
PPARγ	Peroxisome Proliferator-Activated Receptor Gamma
RCR	Respiratory Control Ratio
ROS	Reactive Oxygen Species
RT-PCR	Reverse Transcription Polymerase Chain Reaction
SIRT1	Sirtuin 1
SIRT3	Sirtuin 3
Slc25a5	Solute Carrier Family 25 Member 5
ssEM	Serial Section Electron Microscopy
TCC	Trigeminocervical Complex
TEM	Transmission Electron Microscopy
TFAM	Transcription Factor A Mitochondrial
TG	Trigeminal Ganglion
TNC	Trigeminal Nucleus Caudalis
Tomm34	Translocase of Outer Mitochondrial Membrane 34
Tomm6	Translocase of Outer Mitochondrial Membrane 6
Tomm70	Translocase of Outer Mitochondrial Membrane 70
TRPA1	Transient Receptor Potential Ankyrin 1
TRPV1	Transient Receptor Potential Vanilloid 1
VDAC1	Voltage- Dependent Anion Channel 1
